# Supplementary material for: Psychometric properties of the Generalised Anxiety Disorder Dimensional Scale in an Australian sample
Source: PLoS One. 2023 Jun 6;18(6):e0286634. doi: 10.1371/journal.pone.0286634 (PMC10243613; doi:10.1371/journal.pone.0286634)
Supplement: S1 File — (DOCX) [file pone.0286634.s001.docx]

## Key Statistical Output

**Demographic Data**

| **What is your gender?** | | | | | |
| --- | --- | --- | --- | --- | --- |
|  | | Frequency | Percent | Valid Percent | Cumulative Percent |
| Valid | Male | 73 | 24.9 | 24.9 | 24.9 |
|  | Female | 213 | 72.7 | 72.7 | 97.6 |
|  | Other / prefer not to say | 7 | 2.4 | 2.4 | 100.0 |
|  | Total | 293 | 100.0 | 100.0 |  |

| **What is your current marital status?** | | | | | |
| --- | --- | --- | --- | --- | --- |
|  | | Frequency | Percent | Valid Percent | Cumulative Percent |
| Valid | Single | 186 | 63.5 | 63.5 | 63.5 |
|  | Married | 53 | 18.1 | 18.1 | 81.6 |
|  | De Facto | 38 | 13.0 | 13.0 | 94.5 |
|  | Divorced | 11 | 3.8 | 3.8 | 98.3 |
|  | Widowed | 1 | .3 | .3 | 98.6 |
|  | Separated | 4 | 1.4 | 1.4 | 100.0 |
|  | Total | 293 | 100.0 | 100.0 |  |

| **What is your current employment status?** | | | | | |
| --- | --- | --- | --- | --- | --- |
|  | | Frequency | Percent | Valid Percent | Cumulative Percent |
| Valid | Working part time | 83 | 28.3 | 28.3 | 28.3 |
|  | Working full time | 80 | 27.3 | 27.3 | 55.6 |
|  | Unemployed | 13 | 4.4 | 4.4 | 60.1 |
|  | Studying | 101 | 34.5 | 34.5 | 94.5 |
|  | Retired | 2 | .7 | .7 | 95.2 |
|  | Full time carer | 2 | .7 | .7 | 95.9 |
|  | Other | 12 | 4.1 | 4.1 | 100.0 |
|  | Total | 293 | 100.0 | 100.0 |  |

| **Please indicate your highest level of education.** | | | | | |
| --- | --- | --- | --- | --- | --- |
|  | | Frequency | Percent | Valid Percent | Cumulative Percent |
| Valid | School certificate | 29 | 9.9 | 9.9 | 9.9 |
|  | Trade certificate | 17 | 5.8 | 5.8 | 15.7 |
|  | Higher school certificate | 140 | 47.8 | 47.8 | 63.5 |
|  | Bachelor Degree | 59 | 20.1 | 20.1 | 83.6 |
|  | Postgraduate degree | 41 | 14.0 | 14.0 | 97.6 |
|  | Doctorate | 7 | 2.4 | 2.4 | 100.0 |
|  | Total | 293 | 100.0 | 100.0 |  |

| **Please indicate your country of origin. - Selected Choice** | | | | | |
| --- | --- | --- | --- | --- | --- |
|  | | Frequency | Percent | Valid Percent | Cumulative Percent |
| Valid | Australia | 226 | 77.1 | 77.1 | 77.1 |
|  | New Zealand | 2 | .7 | .7 | 77.8 |
|  | Asia | 11 | 3.8 | 3.8 | 81.6 |
|  | Europe | 4 | 1.4 | 1.4 | 82.9 |
|  | UK | 7 | 2.4 | 2.4 | 85.3 |
|  | North America | 2 | .7 | .7 | 86.0 |
|  | South America | 2 | .7 | .7 | 86.7 |
|  | Middle East | 11 | 3.8 | 3.8 | 90.4 |
|  | Africa | 3 | 1.0 | 1.0 | 91.5 |
|  | Other | 25 | 8.5 | 8.5 | 100.0 |
|  | Total | 293 | 100.0 | 100.0 |  |

| **Descriptive Statistics** | | | | | |
| --- | --- | --- | --- | --- | --- |
|  | N | Minimum | Maximum | Mean | Std. Deviation |
| Please indicate your age (in years). | 293 | 18 | 76 | 28.31 | 12.111 |
| Valid N (listwise) | 293 |  |  |  |  |

**Descriptive Statistics**

| **Descriptives** | | | | |
| --- | --- | --- | --- | --- |
|  | | | Statistic | Std. Error |
| GAD-D Total | Mean | | 12.6007 | .54076 |
|  | 95% Confidence Interval for Mean | Lower Bound | 11.5364 |  |
|  |  | Upper Bound | 13.6650 |  |
|  | 5% Trimmed Mean | | 12.0017 |  |
|  | Median | | 11.0000 |  |
|  | Variance | | 85.679 |  |
|  | Std. Deviation | | 9.25630 |  |
|  | Minimum | | .00 |  |
|  | Maximum | | 40.00 |  |
|  | Range | | 40.00 |  |
|  | Interquartile Range | | 13.00 |  |
|  | Skewness | | .791 | .142 |
|  | Kurtosis | | .233 | .284 |
| GAD-7 Total | Mean | | 8.0648 | .31726 |
|  | 95% Confidence Interval for Mean | Lower Bound | 7.4404 |  |
|  |  | Upper Bound | 8.6893 |  |
|  | 5% Trimmed Mean | | 7.8333 |  |
|  | Median | | 7.0000 |  |
|  | Variance | | 29.492 |  |
|  | Std. Deviation | | 5.43069 |  |
|  | Minimum | | .00 |  |
|  | Maximum | | 21.00 |  |
|  | Range | | 21.00 |  |
|  | Interquartile Range | | 8.00 |  |
|  | Skewness | | .630 | .142 |
|  | Kurtosis | | -.375 | .284 |
| PDSS Total | Mean | | 3.7099 | .26907 |
|  | 95% Confidence Interval for Mean | Lower Bound | 3.1803 |  |
|  |  | Upper Bound | 4.2395 |  |
|  | 5% Trimmed Mean | | 3.2330 |  |
|  | Median | | 2.0000 |  |
|  | Variance | | 21.213 |  |
|  | Std. Deviation | | 4.60581 |  |
|  | Minimum | | .00 |  |
|  | Maximum | | 24.00 |  |
|  | Range | | 24.00 |  |
|  | Interquartile Range | | 7.00 |  |
|  | Skewness | | 1.345 | .142 |
|  | Kurtosis | | 1.540 | .284 |

**Assumption Testing: Normality and Outliers**

**GAD-D Total**


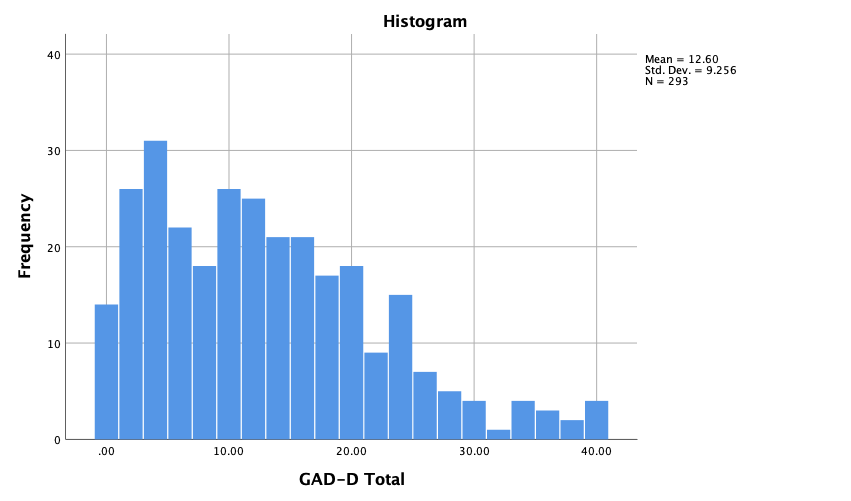


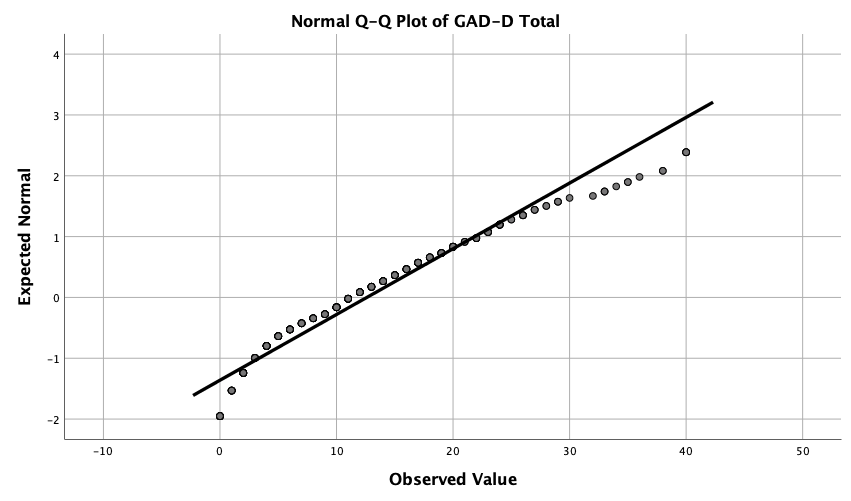


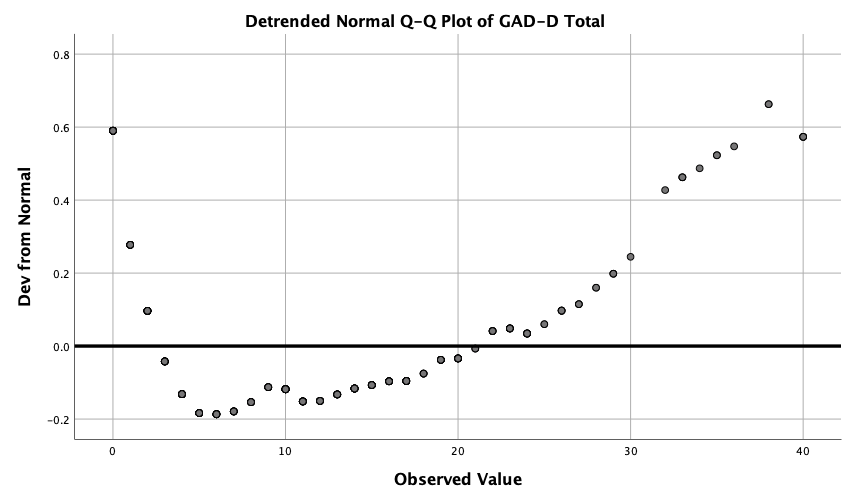


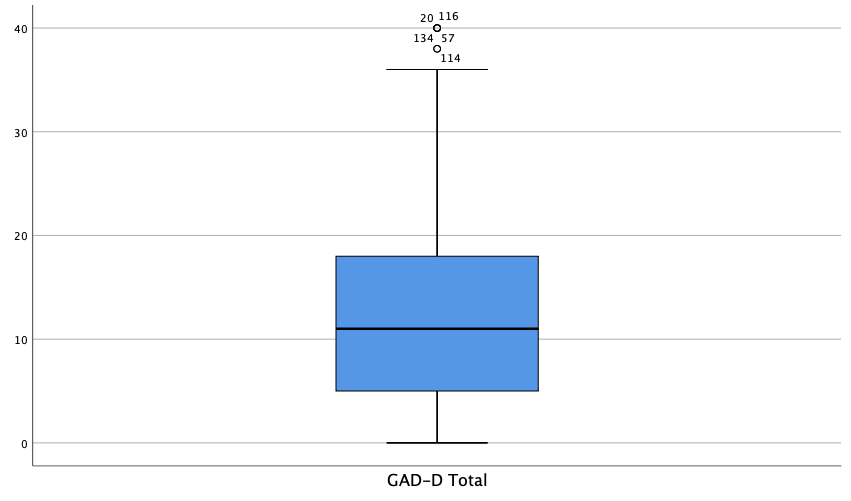


**GAD-7 Total**


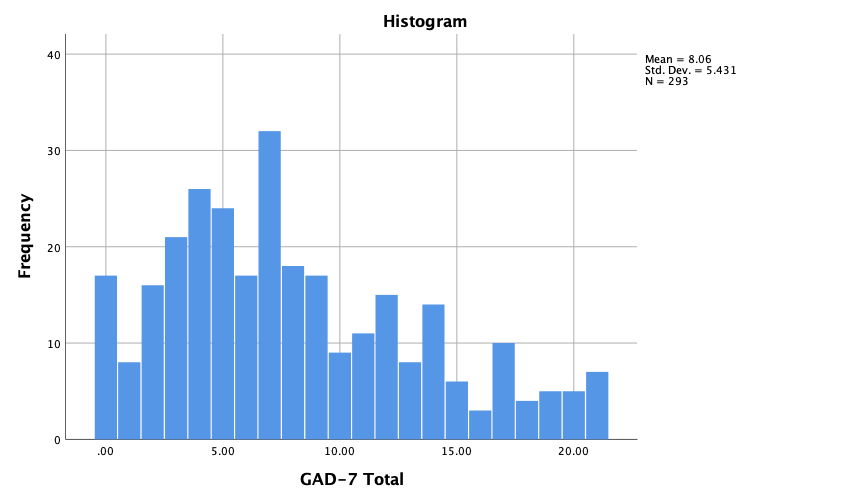


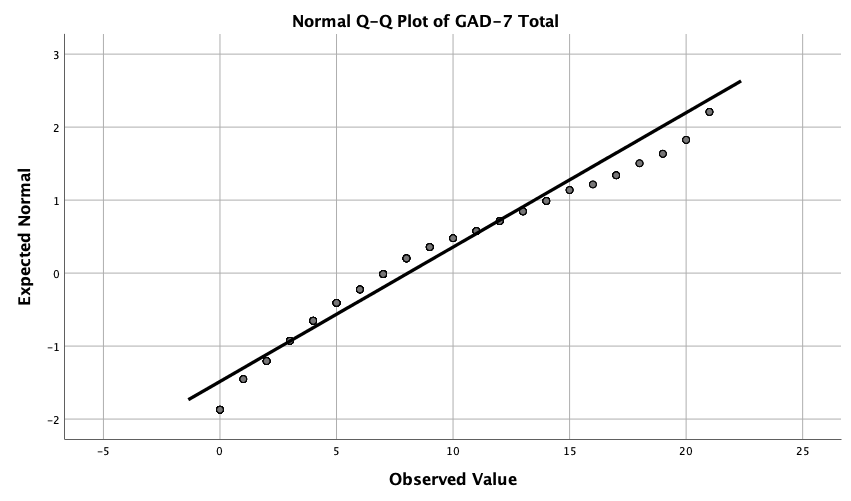


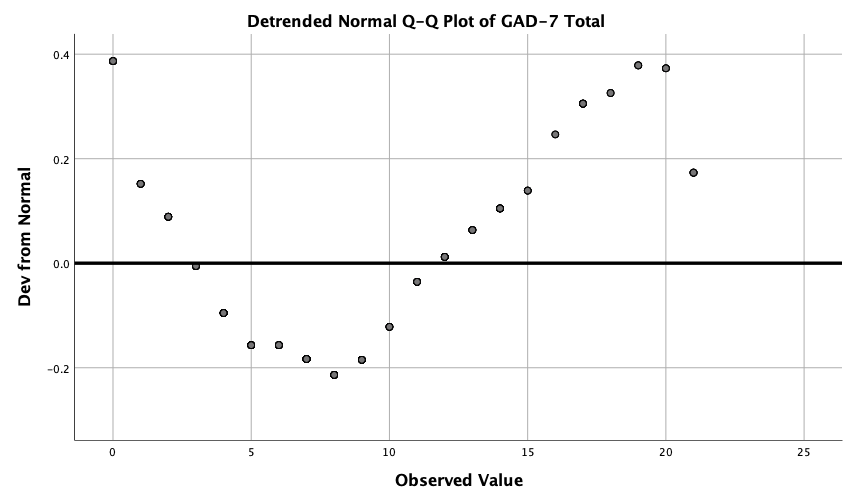


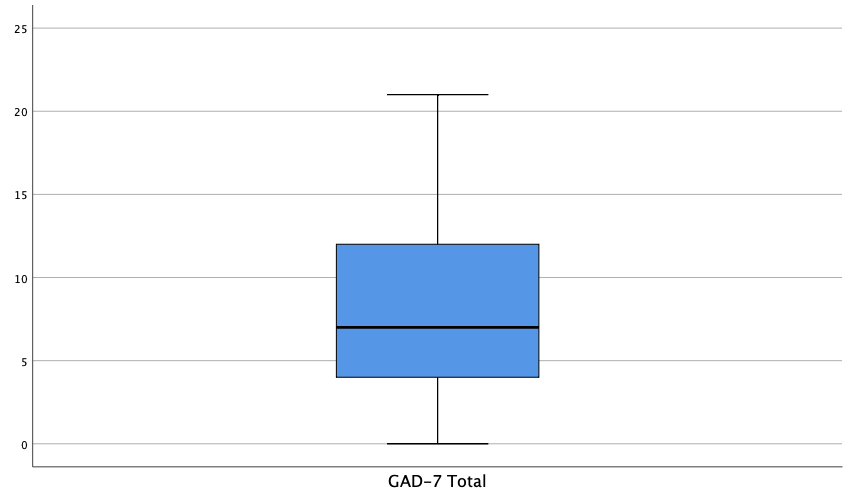


**PDSS Total**


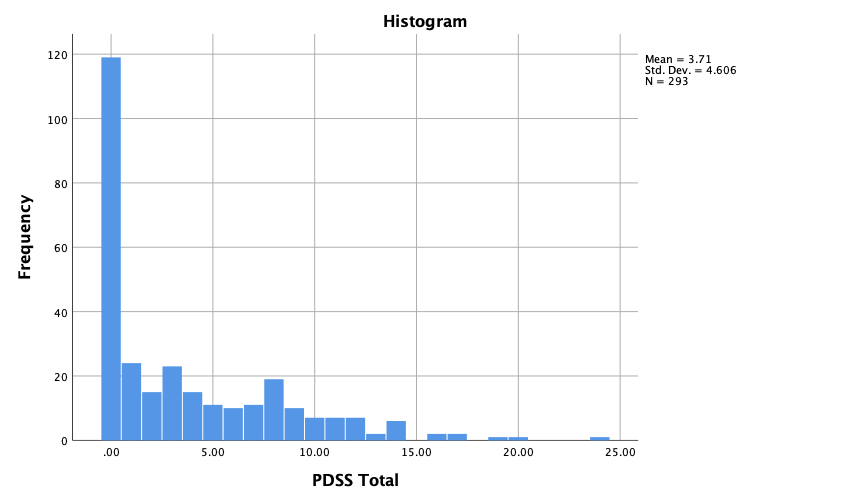


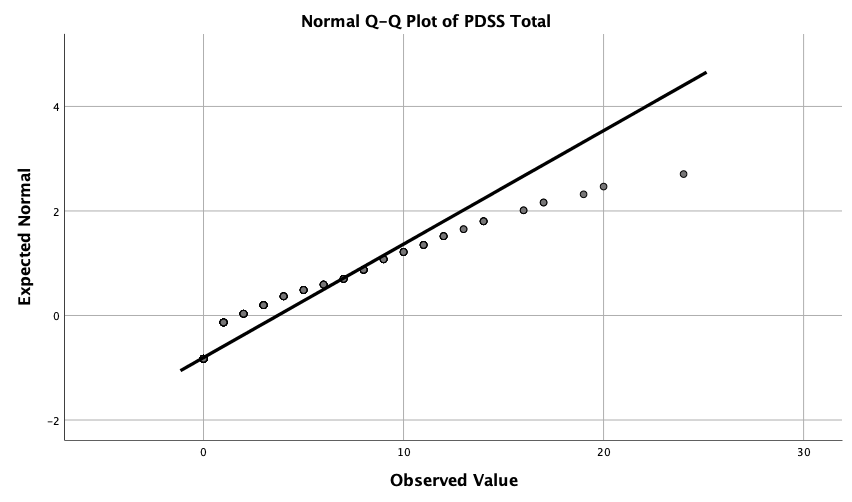


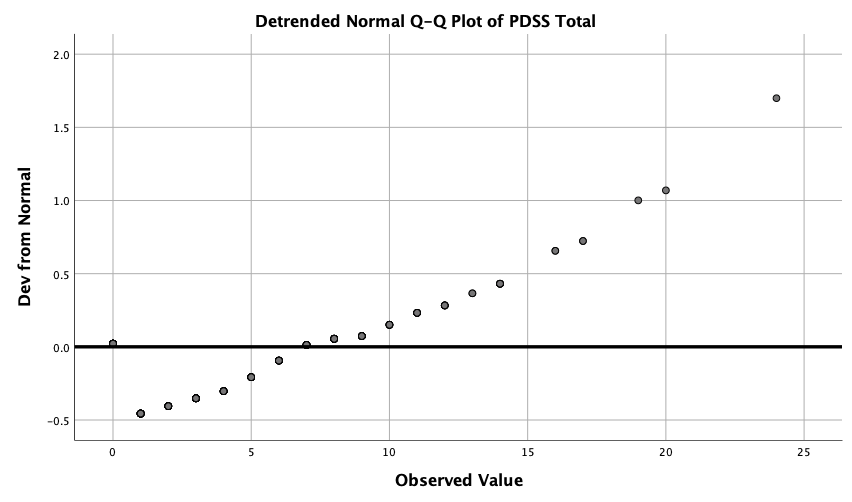


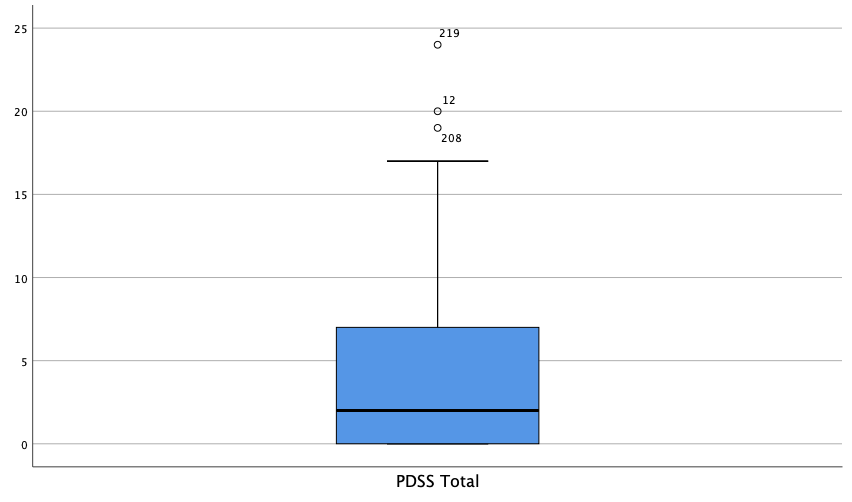


**Internal Consistency: Part 1**

**Scale: GAD-D**

| **Case Processing Summary** | | | |
| --- | --- | --- | --- |
|  | | N | % |
| Cases | Valid | 293 | 100.0 |
|  | Excluded^a^ | 0 | .0 |
|  | Total | 293 | 100.0 |

| a. Listwise deletion based on all variables in the procedure. |
| --- |

| **Reliability Statistics** | |
| --- | --- |
| Cronbach's Alpha | N of Items |
| .935 | 10 |

**Scale: GAD-7**

| **Case Processing Summary** | | | |
| --- | --- | --- | --- |
|  | | N | % |
| Cases | Valid | 293 | 100.0 |
|  | Excluded^a^ | 0 | .0 |
|  | Total | 293 | 100.0 |

| a. Listwise deletion based on all variables in the procedure. |
| --- |

| **Reliability Statistics** | |
| --- | --- |
| Cronbach's Alpha | N of Items |
| .914 | 7 |

**Scale: PDSS-SR**

| **Case Processing Summary** | | | |
| --- | --- | --- | --- |
|  | | N | % |
| Cases | Valid | 293 | 100.0 |
|  | Excluded^a^ | 0 | .0 |
|  | Total | 293 | 100.0 |

| a. Listwise deletion based on all variables in the procedure. |
| --- |

| **Reliability Statistics** | |
| --- | --- |
| Cronbach's Alpha | N of Items |
| .918 | 7 |

**Internal Consistency: Part 2**

**Scale: GAD-D (Part 2)**

| **Case Processing Summary** | | | |
| --- | --- | --- | --- |
|  | | N | % |
| Cases | Valid | 21 | 100.0 |
|  | Excluded^a^ | 0 | .0 |
|  | Total | 21 | 100.0 |

| a. Listwise deletion based on all variables in the procedure. |
| --- |

| **Reliability Statistics** | |
| --- | --- |
| Cronbach's Alpha | N of Items |
| .905 | 10 |

**Test-Retest Reliability**

| **Case Processing Summary** | | | |
| --- | --- | --- | --- |
|  | | N | % |
| Cases | Valid | 21 | 100.0 |
|  | Excluded^a^ | 0 | .0 |
|  | Total | 21 | 100.0 |

| a. Listwise deletion based on all variables in the procedure. |
| --- |

| **Intraclass Correlation Coefficient** | | | | | | | |
| --- | --- | --- | --- | --- | --- | --- | --- |
|  | Intraclass Correlation^b^ | 95% Confidence Interval | | F Test with True Value 0 | | | |
|  |  | Lower Bound | Upper Bound | Value | df1 | df2 | Sig |
| Single Measures | .847^a^ | .659 | .935 | 11.557 | 20 | 20 | .000 |
| Average Measures | .917^c^ | .795 | .966 | 11.557 | 20 | 20 | .000 |

| Two-way mixed effects model where people effects are random and measures effects are fixed. |
| --- |
| a. The estimator is the same, whether the interaction effect is present or not. |
| b. Type A intraclass correlation coefficients using an absolute agreement definition. |
| c. This estimate is computed assuming the interaction effect is absent, because it is not estimable otherwise. |

**Comparing GAD-D scores on test-retest participants vs part 1 only participants**

**T-Test**

| **Group Statistics** | | | | | |
| --- | --- | --- | --- | --- | --- |
|  | Time 2 Y‎/N | N | Mean | Std. Deviation | Std. Error Mean |
| GAD-D Total | Did not complete | 272 | 12.7059 | 9.39586 | .56971 |
|  | Did complete | 21 | 11.2381 | 7.25193 | 1.58250 |

| **Independent Samples Test** | | | | | | | |
| --- | --- | --- | --- | --- | --- | --- | --- |
|  | | Levene's Test for Equality of Variances | | t-test for Equality of Means | | |  |
|  |  | F | Sig. | t | df | Sig. (2-tailed) |  |
|  |  |  |  |  |  |  |  |
| GAD-D Total | Equal variances assumed | 2.421 | .121 | .700 | 291 | .485 |  |
|  | Equal variances not assumed |  |  | .873 | 25.488 | .391 |  |

**R Code and Output**

**Comparing Correlations to Establish Discriminant Validity**

# Load data from SPSS file

library(haven)

Part1 <- read_sav("*filepath/*Part 1 Final.sav")

View(Part1)

library(cocor)

# Calculate correlations between variables

cor(Part1$GADD_Total, Part1$GAD7_Total, method = "spearman")

cor(Part1$GADD_Total, Part1$PDSS_Total, method = "spearman")

cor(Part1$GAD7_Total, Part1$PDSS_Total, method = "spearman")

# Compare correlations

cocor.dep.groups.overlap(0.7696898, 0.6238118 , 0.6824998, 293, alternative = "two.sided", test = "all", alpha = 0.05, conf.level = 0.95, null.value = 0,data.name = NULL, var.labels = NULL, return.htest = FALSE)

**Results**

Results of a comparison of two overlapping correlations based on dependent groups

Comparison between r.jk = 0.7697 and r.jh = 0.6238

Difference: r.jk - r.jh = 0.1459

Related correlation: r.kh = 0.6825

Group size: n = 293

Null hypothesis: r.jk is equal to r.jh

Alternative hypothesis: r.jk is not equal to r.jh (two-sided)

Alpha: 0.05

pearson1898: Pearson and Filon's z (1898)

z = 4.5642, p-value = 0.0000

Null hypothesis rejected

hotelling1940: Hotelling's t (1940)

t = 4.9957, df = 290, p-value = 0.0000

Null hypothesis rejected

williams1959: Williams' t (1959)

t = 4.9055, df = 290, p-value = 0.0000

Null hypothesis rejected

olkin1967: Olkin's z (1967)

z = 4.5642, p-value = 0.0000

Null hypothesis rejected

dunn1969: Dunn and Clark's z (1969)

z = 4.8239, p-value = 0.0000

Null hypothesis rejected

hendrickson1970: Hendrickson, Stanley, and Hills' (1970) modification of Williams' t (1959)

t = 4.9957, df = 290, p-value = 0.0000

Null hypothesis rejected

steiger1980: Steiger's (1980) modification of Dunn and Clark's z (1969) using average correlations

z = 4.7937, p-value = 0.0000

Null hypothesis rejected

meng1992: Meng, Rosenthal, and Rubin's z (1992)

z = 4.7779, p-value = 0.0000

Null hypothesis rejected

95% confidence interval for r.jk - r.jh: 0.1701 0.4066

Null hypothesis rejected (Interval does not include 0)

hittner2003: Hittner, May, and Silver's (2003) modification of Dunn and Clark's z (1969) using a backtransformed average Fisher's (1921) Z procedure

z = 4.7631, p-value = 0.0000

Null hypothesis rejected

zou2007: Zou's (2007) confidence interval

95% confidence interval for r.jk - r.jh: 0.0853 0.2123

Null hypothesis rejected (Interval does not include 0)

**Confirmatory Factor Analysis**

# First CFA

library(lavaan)

# Create a single factor using all scale items

gadd <- 'f =~ GADD_1 + GADD_2 + GADD_3 + GADD_4 + GADD_5 + GADD_6 + GADD_7 + GADD_8 + GADD_9 + GADD_10'

# Fit model and output summary of indices

onefactor <- cfa(gadd, data = Part1, estimator = "WLSMV")

summary(onefactor, fit.measures = TRUE)

# Generate modification indices

mi <- modindices(onefactor)

Results

Model Test User Model:

Standard Robust

Test Statistic 24.600 116.441

Degrees of freedom 35 35

P-value (Chi-square) 0.905 0.000

Comparative Fit Index (CFI) 0.895

Tucker-Lewis Index (TLI) 0.865

RMSEA 0.089

90 Percent confidence interval - lower 0.072

90 Percent confidence interval - upper 0.107

P-value RMSEA <= 0.05 0.000

# Second CFA

**#** Insert local dependency between items 6 & 7 in model

gadd2 <- 'f =~ GADD_1 + GADD_2 + GADD_3 + GADD_4 + GADD_5 + GADD_6 + GADD_7 + GADD_8 + GADD_9 + GADD_10

GADD_6 ~~ GADD_7'

onefactor2 <- cfa(gadd2, data = Part1, estimator = "WLSMV")

summary(onefactor2, fit.measures = TRUE)

mi2 <- modindices(onefactor2)

Results

Model Test User Model:

Standard Robust

Test Statistic 18.441 90.133

Degrees of freedom 34 34

P-value (Chi-square) 0.986 0.000

Comparative Fit Index (CFI) 0.927

Tucker-Lewis Index (TLI) 0.904

Root Mean Square Error of Approximation:

RMSEA 0.075

90 Percent confidence interval - lower 0.057

90 Percent confidence interval - upper 0.094

P-value RMSEA <= 0.05 0.015

**Chi Square Test to Compare Part 1 and Part 2 on Gender**

table(Part1$Gender, Part1$Time2)

chisq.test(Part1$Gender, Part1$Time2, correct=TRUE, simulate.p.value = TRUE)

Results

data: Part1$Gender and Part1$Time2

X-squared = 0.66685, df = NA, p-value = 0.7941

**Chi Square Test to Compare Part 1 and Part 2 on Education Level**

table(Part1$Education, Part1$Time2)

chisq.test(Part1$Education, Part1$Time2, correct=TRUE)

Results

data: Part1$Education and Part1$Time2

X-squared = 1.884, df = 5, p-value = 0.865
