# Supplementary material for: Psychometric properties of the Generalised Anxiety Disorder Dimensional Scale in an Australian sample
Source: PLoS One. 2023 Jun 6;18(6):e0286634. doi: 10.1371/journal.pone.0286634 (PMC10243613; doi:10.1371/journal.pone.0286634)
Supplement: Attachment — Submitted filename: GAD-D Response to reviewers_FINAL .docx [file pone.0286634.s002.docx]

Reviewer 1

1- Introduction: It is very long, tedious and contains unnecessary information.

The authors thank the reviewer for this feedback. The introduction has now been revised to increase succinctness and clarity. Please see pages 3-5 of the manuscript.

2- This questionnaire assess Anxiety Disorder but there is no mention of having psychiatric disorders, taking certain medications, etc. in patients. Only demographic characteristic has been presented.

The authors thank the reviewer for this feedback. As a non-clinical community sample has been utilised, psychiatric history was not sought from participants. This has now been clearly referenced. Please see page 6 of the manuscript.

3- Willing to participation is not as inclusion criteria

The authors thank the reviewer for this feedback. The authors have reviewed the manuscript to ensure that willingness to participate has not been included as an inclusion criteria.

4- In construct validity, why Exploratory Factor Analysis (EFA) was not done?

The authors thank the reviewer for this query. Given previous psychometric evaluations of the DSM 5 Dimensional Scales internationally, confirmatory factor analysis was deemed most appropriate to undertake in this study. Importantly, this approach is also consistent with existing literature, including Lebeau et al., 2012, Beesdo-Baum et al., 2012, Macfarlane et al., 2020 and Russell et al., 2020. This has now also been referenced in the manuscript. Please see page 7.

5- Discriminate validity indicates how well an instrument differentiates between different groups who differ in some characteristics. In this study in abstract, there is divergent and in manuscript is discriminate validity. "The correlation between the GAD-D and GAD-7 was significantly greater than the correlation between the GAD-D and PDSS-SR, z(290) = 4.79 (p < .001, two-tailed), providing evidence for discriminate validity". What is the meaning of authors of z(290) = 4.79? Is that correlation between GAD-D and PDSS-SR?

The authors thank the reviewer for this observation. Incorrect reference to divergent validity has now been removed. Please see page 2 of the manuscript.

6- The manuscript repeatedly mentions Part 1 and 2 and Time 1 and 2. If time 1 and 2 referred to test retest time reliability, what is the meaning of parts 1 and 2?

The authors thank the reviewer for this observation. All references to Time 1 and Time 2 have now been changed to Part 1 and Part 2 respectively. This has been changed throughout the manuscript, highlighted in red.

7- The interval time for assess test-retest reliability is not mentioned.

This has now been included. Please see page 7 of the manuscript.

8- The manner of the method section is chaotic.

The authors thank the reviewer for this feedback. The method section has now been revised. Please see pages 5-8 of the manuscript.

9- It's mention that" The time between completion of Part 1 and Part 2 was not controlled or monitored". Why? This is very important and critical.

The authors thank the reviewer for this feedback. Whilst completion time between Part 1 and Part 2 was monitored, it was not strictly adhered to by participants. This section has been reworded for clarity. Please see page 14 of the manuscript.

Reviewer 2

N/A
