# Supplementary material for: Psychometric properties of the Generalised Anxiety Disorder Dimensional Scale in an Australian sample
Source: PLoS One. 2023 Jun 6;18(6):e0286634. doi: 10.1371/journal.pone.0286634 (PMC10243613; doi:10.1371/journal.pone.0286634)
Supplement: Attachment — Submitted filename: Response to reviews v3 .docx [file pone.0286634.s003.docx]

Response to Reviewers

Journal Requirements:
Please review your reference list to ensure that it is complete and correct. If you have cited papers that have been retracted, please include the rationale for doing so in the manuscript text, or remove these references and replace them with relevant current references. Any changes to the reference list should be mentioned in the rebuttal letter that accompanies your revised manuscript. If you need to cite a retracted article, indicate the article’s retracted status in the References list and also include a citation and full reference for the retraction notice.

The reference list has now been reviewed for accuracy and completeness. One omitted reference from the reference list has now been added. Please see page 22 of the revised manuscript.

To the authors knowledge, no retracted articles have been included in this manuscript.

Three new references have been added, relevant to reviewer requests. Please see page 22 of the revised manuscript.

Reviewer #3: 
Abstract

“A sample of 293 Australians (72.7% female) aged between 18 and 73 (M = 28.31 years; SD = 12.11 years) were recruited.” a sample was…?

The authors thank the reviewer for identifying this error. This has now been rephrased as follows: ‘A sample of 293 Australians (72.7% female) aged between 18 and 73 (M = 28.31 years; SD = 12.11 years) was recruited.’ Please see page 2 of the revised manuscript.

Introduction

1.“The number of participants required for the study was based on the recommendation of
Tabachnick and Fidell(32), who specified that a minimum of 300 participants is adequate for a
psychometric analysis that includes examination of factor structure.”
I believe the authors could provide more information on the sample size planning procedure. Not just indicate a defined number for the sample. For example, see Kelley, K., & Lai, K. (2018). Confirmatory factor models: Power and accuracy for effects of interest. The Wiley handbook of psychometric testing: A multidisciplinary reference on survey, scale and test development, 113-139.

The authors thank the reviewer for this feedback. A sampling size procedure has now been listed in the manuscript, which suggests adequate sample size used. This is also consistent with COSMIN guidelines (Terwee et al., 2011). Please see page 9 of the revised manuscript.

2. “This resulted in a final sample of 293(< 300) participants.” The final sample is smaller than initially proposed.

Given use of sampling procedure (as recommended above), sample is now considered within appropriate range.

Methods

1. p.8, about the Generalised anxiety disorder dimensional scale “Cronbach’s α in the present study was .94.” I believe this should be reported in the results

The authors thank the reviewer for this feedback. This is now recorded in the results section only. Please see page 10 of the revised manuscript.

Results

It is not explicitly stated in the manuscript whether the analyzed data are available in any public repository. If not, it would be ideal if the authors would do so.

The authors thank the reviewer for highlighting this omission. As the data used for this project was collected as part of a larger body of research still currently in use, data is not currently available in a public repository, though may be requested from the lead investigator. This has now been added to the manuscripts as follows: ‘Data is available from the lead investigator upon reasonable request. ‘ Please see page 7 of the revised manuscript.

Discussion

1.p.13 “To the author’s knowledge,…” to the authors' knowledge?

For clarity of language, this has now been removed. Please see page 13 of the revised manuscript.

2. “Therefore, future studies evaluating the psychometric properties of the GAD-D should attempt to exceed the minimum 300 participants specified by Tabachnick and Fidell(32)” Again, I believe that the authors could make explicit a sampling procedure based on CFA, especially if the authors suggest that a higher n could guarantee a better model fit.

The authors thank the reviewer again for this recommendation. A revised sampling procedure has now been included, which suggests sample utilised is within appropriate range. Please see page 9 of the revised manuscript.

3. "This may suggest that avoidance and escape behaviours are typically observed in individuals that have GAD, despite their exclusion from the DSM-5 criteria. This would align particularly with models of GAD that highlight the maintaining role of avoidance; for example, those proposed by Borkovec (27) and Dugas et al.(42)" Interesting, future proposed research could add something to this point, considering the assessment (self-report) of avoidance and escape behaviors in clinical population having or not the GAD diagnosis.

Thank you for this observation. This has now been highlighted as an area of further research in the manuscript. Please see page 12 of the revised manuscript.

4. “Notably, although all items demonstrated adequate factor loadings in the present study, items 9 and 10 had a notably lower loading onto the general factor than the other items of the scale.” Any of the expressions "notably" may be changed.

Thank you for this observation. Wording has now been changed to the following: ‘Notably, although all items demonstrated adequate factor loadings in the present study, items 9 and 10 had a lower loading onto the general factor than the other items of the scale.’ Please see page 12 of the revised manuscript.

5. "...indicating that the present community sample had a similar or even greater level of GAD pathology than a sample of treatment-seeking individuals." It would not assert that it is GAD pathology if there is no clinical diagnosis to support it, but rather related symptoms or or anxiety problems.

The authors thank the reviewer for highlighting this important distinction. This has now been corrected in the manuscript as follows: ‘indicating that the present community sample had a similar or even greater level of GAD or other anxiety symptomology than a sample of treatment-seeking individuals.’ Please see page 15 of the revised manuscript.

6. "Future research should analyse the sensitivity and specificity of the GAD-D in an Australian sample to establish a cut-off point and allow Australian clinicians to also use the GAD-D as a screening tool". I believe that it should be emphasized that in order to achieve "cut-off points" it is necessary to include in the sample a properly diagnosed clinical population.

The authors thank the reviewer for identifying this important point. This has now been reflected in the manuscript as follows: ‘Future research should analyse the sensitivity and specificity of the GAD-D in an Australian sample to establish a cut-off point and allow Australian clinicians to also use the GAD-D as a screening tool, using a clinical sample of individuals who have received diagnosis via structured interview.’ Please see page 16 of the revised manuscript.

Reviewer #4: 
1. Authors should provide what methods they used for data collection. While authors have mentioned an online questionnaire was used, there is no mention of website or software used for online survey.

The authors thank the reviewer for identifying this omission. This has now been included, as follows: ‘All data was collected via Qualtrics.’ Please see page 7 of the revised manuscript.

2. While 140 (48%) individuals expressed interest in completing Part 2, only 21 participants provided complete data for Part 2. Any specific reason for very small retention? Does authors sent any reminder to those who showed interest for participation in part 2?

The authors thank the reviewer for this feedback. It has now been highlighted in the manuscript (results section) that only one follow up email was sent to participants. Please see pages 9 and 10 of the revised manuscript.

The following has now also been added to the discussion section: ‘This is likely due to only one follow up email being sent to participants who expressed interest in completing this part of the study ‘and ‘Future research may also wish to use more follow up emails to increase participation in Part 2.’ Please see pages 14 and 15 of the revised manuscript.

3. Sample size for retest analysis is too small. I would suggest to follow-up with those who showed interest to participate in part 2. Alternatively, authors may conduct additionally survey, which include at least 5o subjects in retest survey.

The authors thank the reviewer for this feedback. We accept that the sample size for re-test analysis is small. Unfortunately, collecting further data is not a possibility. It is noted that some references indicate that our sample size is broadly within range. For example, with two observations per subject, an ICC greater than 0.5, an α of 0.05, and power of 0.80, 22 participants are required to estimate the value of ICC (Bujang, 2017). This has now been noted in the manuscript. However, given this, we emphasise that assessment of re-test reliability is preliminary in nature within the manuscript. Please see pages 9 and 15 of the revised manuscript.
