# Supplementary material for: Psychometric properties of the Generalised Anxiety Disorder Dimensional Scale in an Australian sample
Source: PLoS One. 2023 Jun 6;18(6):e0286634. doi: 10.1371/journal.pone.0286634 (PMC10243613; doi:10.1371/journal.pone.0286634)
Supplement: Attachment — Submitted filename: PLOS One response to reviewers v3.docx [file pone.0286634.s004.docx]

Response to Reviewers

Reviewer #3: (No Response)

Reviewer #4: I congratulate authors for revising their manuscript incorporating the reviewer comments. However, I still have some minor comments as follow:

1. In the last paragraph of introduction section, author stated that "The following will be investigated: 1) factor structure; 2) reliability; and 3) convergent and discriminant". I think author should use a past tense rather using future tense.

The authors thank the reviewer for this observation. This has now been corrected as follows: ‘The following were investigated: 1) factor structure; 2) reliability; and 3) convergent and discriminant validity.’ Please see page 5 of the manuscript.

2. It is hypothesized that the psychometric properties of this scale will be consistent with ...?

The authors thank the reviewer for this feedback. This has now been corrected to as follows: ‘It was hypothesised that the psychometric properties of this scale will be consistent with previous evaluations (11, 13, 14, 16, 23) . Specifically, the GAD-D is expected to demonstrate a unidimensional factor structure, excellent internal consistency, good test-retest reliability, convergent and discriminant validity.’ Please see page 5 of the manuscript.

3. Please explain GAD-D before using abbreviation.

The authors thank the reviewer for this observation. This has now been corrected. Please see page 4 of the manuscript.

4. This use of this analysis is consistent with that used in the existing literature (16, 17, 30,..)? Please check this sentence

The authors thank the reviewer for this observation. This has now been corrected as follows: ‘The use of this analysis is consistent with that used in the existing literature (16, 17, 30, 31).’ Please see page 7 of the manuscript.

5. Please explain Little’s MCAR test?

The authors thank the reviewer for highlighting this omission. This has now been added as follows: ‘Little’s MCAR test, designed to test for patterns in missing data, indicated that the data was missing completely at random, *χ2*(720) = 678.01, *p* = .87.’ Please see page 9 of the manuscript.

6. More than 50% citations are 10 years older. Please replace some of them with more recent citations.

The authors thank the reviewer for highlighting this. Prior to this feedback, 42 references were included. Of these, 5 have been replaced with references within the last ten years, three have been removed and one has been added. This results in 40 references at this submission. Of these, 15 are 10 years or older, resulting in only 37.5% of references being 10 years or older. Please see page 17 of the manuscript.
